# Healing, Antioxidant and Cytoprotective Properties of *Indigofera truxillensis* in Different Models of Gastric Ulcer in Rats

**DOI:** 10.3390/ijms131114973

**Published:** 2012-11-15

**Authors:** Anderson Luiz-Ferreira, Maira Cola, Victor Barbastefano, Felipe Meira de-Faria, Ana Beatriz A. de Almeida, Elisângela Farias-Silva, Tamara Regina Calvo, Clélia A. Hiruma-Lima, Wagner Vilegas, Alba Regina M. Souza-Brito

**Affiliations:** 1 Department of Biological Sciences, Federal University of Goiás, 75704-020, Catalão, Goiás, Brazil; 2 Department of Structural and Functional Biology, Biology Institute, Campinas University, 13083-865, Campinas, São Paulo, Brazil; E-Mails: vicbio@yahoo.com (V.B.); anabia5@yahoo.com.br (A.B.A.A.); elisfarias3@yahoo.com.br (E.F.-S.); abrito@unicamp.br (A.R.M.S.-B.); 3 Department of Pharmacology, Faculty of Medical Sciences, University of Campinas, 13083-887, Campinas, São Paulo, Brazil; E-Mails: mcola1@yahoo.com.br (M.C.); defaria.felipe@yahoo.com.br (F.M.F.); 4 Department of Organic Chemistry, Institute of Chemistry, São Paulo State University, 14800-900, Araraquara, São Paulo, Brazil; E-Mails: tamaracalvo@yahoo.com.br (T.R.C.); vilegasw@gmail.com (W.V.); 5 Department of Physiology, Institute of Biosciences, São Paulo State University, 18618-000, Botucatu, São Paulo, Brazil; E-Mail: hiruma@ibb.unesp.br

**Keywords:** *Indigofera truxillensis*, gastric ulcer, medicinal plants, antioxidant enzymes

## Abstract

The present study evaluated the antiulcerogenic activity and mechanisms of the aqueous (AqF 100 mg/kg) and ethyl acetate (AcF 50 mg/kg) fractions from *Indigofera truxillensis* leaves. This dose was selected to assess its activity on ulcer healing and its action on gastric acid and mucus secretion, prostaglandin production and antioxidant enzyme activity (superoxide dismutase (SOD), glutathione peroxidase (GSH-Px) and glutathione reductase (GSH-Rd)). Gastric ulcer was induced by absolute ethanol. Antisecretory action, mucus and prostaglandin production, healing and antioxidant enzyme activities were evaluated for both fractions. AqF and AcF significantly inhibited the gastric mucosal damage caused by ethanol. This effect was statistically significant at 100 and 50 mg/kg compared with the vehicle. Neither fraction interfered with gastric secretion. AcF increased the PGE_2_ production, and both fractions increased mucus production. l-NAME did not alter the gastroprotection exerted by the fractions, but *N*-ethylmaleimide attenuated only AcF. In the ischemia/reperfusion model both fractions inhibited the mucosal damage. AcF increased SOD, GSH-Px and GSH-Rd activity, but AqF increased only SOD and GSH-Px. In the acetic acid-induced ulcer model AcF only accelerated ulcer healing. These results showed that *Indigofera truxillensis* acted as a gastroprotective agent, stimulating protective factors and antioxidants enzymes.

## 1. Introduction

The genus *Indigofera*, one of the six largest genera of the Leguminosae, contains approximately 700 herbaceous and bushy species and occurs in the state of São Paulo and in north and northeastern Brazil [1]. *Indigofera truxillensis* is typical of the ‘cerrado’, Brazilian’s savannah like region, known by the popular name of ‘anileira’. Furthermore, the aerial parts of *I. truxillensis* are used in folk medicine for the treatment of gastric pain and disorders [2,3]. Apart these medicinal uses, pharmacological studies showed antimycobacterial and antiulcerogenic activities of another species of the same Genus, *I. suffruticosa*[4,5].

Phytochemical studies carried out with *I. truxillensis* revealed an abundant amount of several compounds, including kaempferol 3-*O*-α-l-rhamnopyranoside and kaempferol 7-*O*-α-l-rhamnopyranoside, kaempferol 3-*O*-α-l-rhamnopyranoside-7-*O*-α-1-rhamnopyranoside and kaempferol 3-*O*-α-l-arabinopyranoside- 7-*O*-α-l-rhamnopyranoside, as well as the alkaloids indigo and indirubin [6]. Recently, we have demonstrated that the alkaloid indigo from *I. truxillensis* modulates antioxidant enzymes in the rat and markedly inhibits gastric mucosal lesions induced by ethanol 70% [7].

The present work was carried out to investigate the possible antiulcerogenic effect of the ethyl acetate (AcF) and aqueous (AqF) fractions from the leaves of *I. truxillensis* on acute and subacute experimental models of gastric ulcers in rodents. In addition, antioxidant enzymes (superoxide dismutase (SOD), glutathione peroxidase (GSH-Px) and glutathione reductase (GSH-Rd)), mucus secretion, prostaglandin production and acid secretion were evaluated as possible mechanisms of action of the fractions.

## 2. Results and Discussion

### 2.1. Analyses of Secondary Metabolites

The analyses of the aqueous fraction (AqF) showed the presence of flavonoids (52%), glycerolipids (43%) and alkaloids (5%), while the ethyl acetate fraction (AcF) contained flavonoids (28%), glycerolipids (48%) and alkaloids (24%).

### 2.2. Ethanol-Induced Gastric Lesions

The oral doses (50, 100 and 200 mg/kg) were initially used to establish a general profile of the antiulcerogenic activity of both AqF and AcF (Table 1). Both fractions protected the mucosa against the damage caused by ethanol at all the tested doses. However, the AcF fraction presented the same profile of gastroprotection at all the doses tested (50, 100 and 200 mg/kg), but with no significant statistical difference between them. Therefore, in the other assays, we used the lowest effective dose (50 mg/kg). In the AqF fraction, the lowest effective dose was 100 mg/kg, which determined its subsequent use.

A complex series of morphological and functional changes occurs in gastric tissues after exposure to various ulcerogenic agents. The administration of absolute ethanol produces numerous gastric mucosal lesions, a decrease in gastric blood flow, generation of reactive oxygen species and an increase in inflammatory mediators, including IL-1β and TNF-α [8].

The balance between the therapeutic and the toxicological effects of a drug is an important parameter when assessing its applicability regarding the pharmacological action [9]. Cola-Miranda *et al.*[3] showed that the groups treated with *Indigofera truxillensis* did not show significant differences in body weight development or in organ weight between the groups studied. In addition, no macroscopic abnormalities or mortality was observed during the 14-day study. In addition, we evaluated the cytotoxicity of the fractions (2, 4, 6, 8 and 10 mg/mL) used in this work in V79 cells by the neutral red assay, which showed no toxicity (data not shown).

*Indigofera truxillensis* has been documented to contain the alkaloid indigo as its main compound [6]. Farias-Silva *et al.*[7] showed that indigo partially protects the gastric mucosa against ethanol-induced DNA damage. Since the AcF from *Indigofera truxillensis* has been found to contain indigo as its constituent, the mucosal protection against ethanol of the AcF (50, 100 or 200 mg/kg) was evaluated. AcF fractions protected the gastric mucosa at the three doses tested without any statistically significant difference between them. Thus, the remaining tests were conducted with the most effective dose for AqF (100 mg/kg) and the lowest effective dose for AcF (50 mg/kg). The AqF fraction was more effective at 100 mg/kg when compared with doses of 50 and 200 mg/kg. The AqF fraction presents 52% flavonoids in its composition, which partially explains its behavior in this model. Low concentrations of the flavonoid present antioxidant activity whereas higher concentrations of the flavonoid decrease this activity [10]. Our results are in accordance with those obtained by Cola-Miranda *et al.*[3] who evaluated the antiulcerogenic action of the methanolic extract from *I. truxillensis*.

### 2.3. Gastric Secretion in Lesions Induced by Pylorus Ligature

To evaluate the biochemical parameters from the gastric content, rats were subjected to pylorus ligature and treatment with AqF and AcF from *I. truxillensis*. The results obtained did not change the pH. However, the animals treated with AcF (50 mg/kg) showed a diminished gastric volume (Table 2).

Gastric acid secretion is a phylogenetically old function that developed about 500 million years ago [11]. It is regulated by biological agents, produced and released by enteroendocrine cells and neurons, as well as by exogenously administered substances and infections. When gastric secretion is excessive, it can lead to peptic ulcer disease [12]. However, excessive acid secretion is not, in many cases, the main player in the genesis of peptic ulcer [13]. Corroborating with Cola-Miranda *et al.*[3] in the pylorus ligature model, both fractions were not capable of inhibiting the acid secretion. It is also necessary to consider that the decrease in acid secretion can interfere with the absorption of certain nutrients and medications and may also lead to a predisposition to enteric infection [14].

### 2.4. Prostaglandin Production Determination

**Figure 1:**
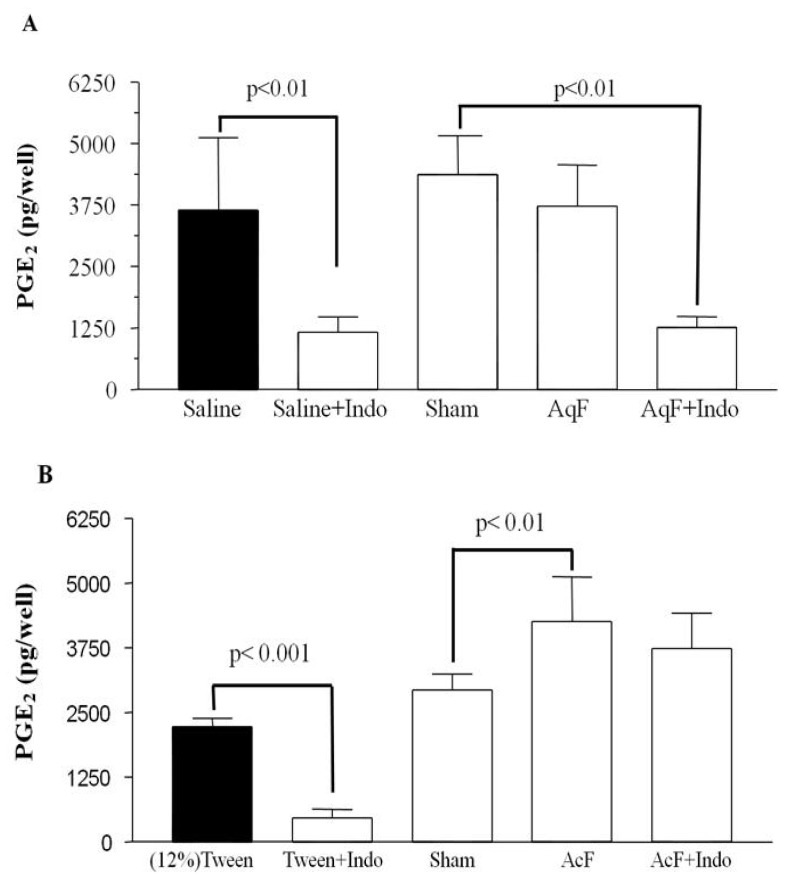
Effect of orally administered (**A**) AqF (100 mg/kg) and (**B**) AcF (50 mg/kg) from *Indigofera truxillensis* and indomethacin on gastric prostaglandin E_2_ (PGE_2_) production in rats. The results are reported as the mean ± SD. ANOVA followed by Tukey’s test. *p* < 0.01 compared to the corresponding vehicle group.

Figure 1A shows that AqF (100 mg/kg) was not able to maintain the levels of PGE_2_. On the other hand, AcF (50 mg/kg) increased and maintained a high PGE_2_ level despite the administration of indomethacin (Figure 1B). AcF was able to promote a sustained increase in PGE_2_ levels, which is vital to the integrity of the gastric mucosa. Furthermore, the combination of AcF and NSAID was not capable of reducing the levels of PGE_2_ release in a significant manner.

The gastric mucosa is continuously exposed to noxious substances and has specific defense mechanisms for maintaining its structural integrity [15]. The epithelial surface secretes a barrier consisting of water, mucin bicarbonate and prostaglandins [15].

Prostaglandins, particularly PGE_2_, play an important role in modulating the mucosal integrity by the regulation of gastro-duodenal bicarbonate secretion [15,16]. We observed that AqF (100 mg/kg) treatment was unable to increase PGE_2_ levels in the gastric mucosa when compared to the sham group. Phenol compounds such as flavonoids have a dual effect on prostaglandin biosynthesis; low concentrations stimulate and high concentrations inhibit it [17]. Probably, the dose used may inhibit PGE_2_ secretion, exerting its gastroprotective action through a distinct mechanism. AcF (50 mg/kg) was able to increase PGE_2_ levels when compared to the sham group, these levels were kept even in the presence of indomethacin. Toma *et al.*[18] showed that alkaloids are capable of increasing prostaglandin and mucus production. Prostaglandin stimulates mucus secretion and also protects the gastric mucosa against noxious agents such as ethanol and indomethacin [16].

### 2.5. Determination of the Gastric Mucus Content

**Figure 2:**
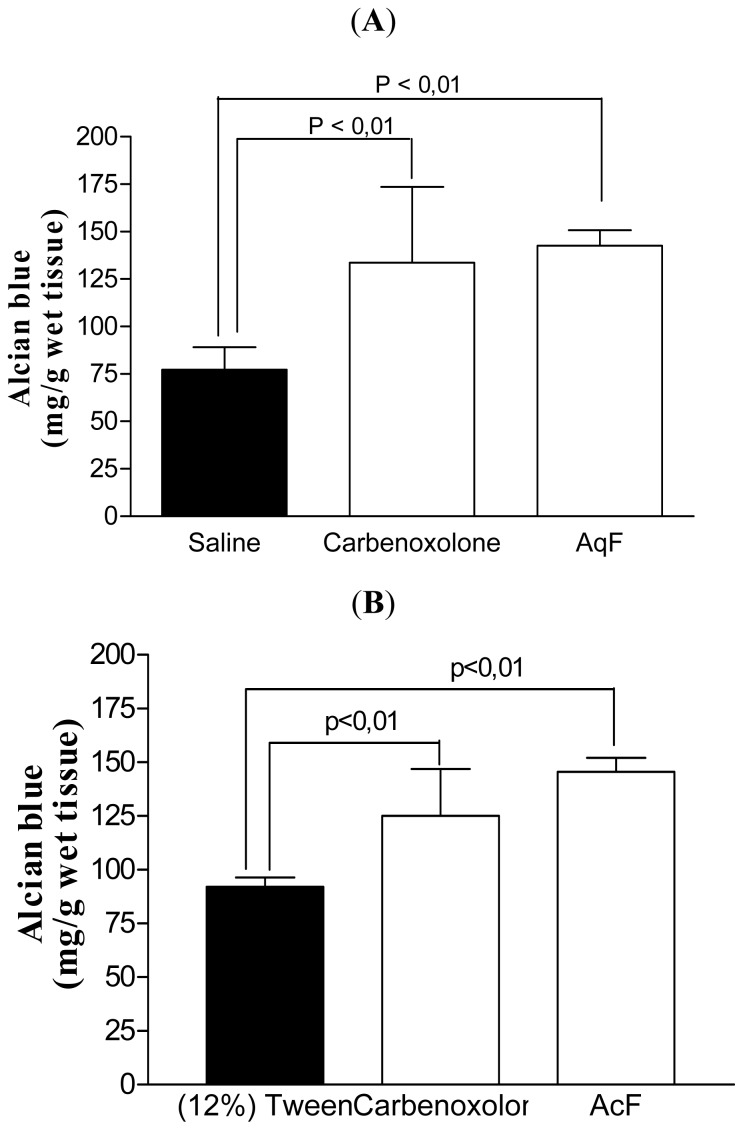
Effects of oral carbenoxolone (200 mg/kg), (**A**) AqF (100 mg/kg) or (**B**) AcF (50 mg/kg) obtained from *Indigofera truxillensis* on the production of adherent gastric mucus (measured as the amount of bound alcian blue) in pylorus-ligated rats. The results are the mean ± SD. ANOVA followed by Tukey’s test (*p* < 0.01).

Still looking for a possible mechanism for the increase in gastric mucosa protective factors, we investigated the effects of AcF (50 mg/kg) and AqF (100 mg/kg) on mucus production. Pretreatment with both fractions and carbenoxolone (200 mg/kg) significantly increased the amount of adherent mucus in the gastric mucosa when compared to the control group (Figure 2A,B).

The mucus secretion, another defense mechanism for the maintenance of the gastric mucosa, covers the gastrointestinal tract, including the stomach, protecting the mucosa against damage [19]. Pretreatment with carbenoxolone (200 mg/kg), AqF (100 mg/kg) or AcF (50 mg/kg) significantly increased the amount of adherent mucus in the gastric mucosa when compared to the control group. A range of environmental stimuli can alter the rate of mucin release, including prostaglandin [16] and nitric oxide (NO) [20]. Considering that AqF (100 mg/kg) was unable to increase prostanglandin production, this increase in mucus can be a result of NO. In fact, the expression of mucins by mucosal epithelial cells *in vitro* can be directly upregulated by NO [21]. On the other hand, the increase promoted by AcF (50 mg/kg) can be attributed to a PGE_2_ increase promoted by the fraction. This gastric mucus increase is one of the antiulcerogenic mechanisms involved with the antiulcer effects of this fraction.

### 2.6. Determination of the Role of Nitric Oxide (NO) and Sulfhydryl Compounds (SH) in Gastric Protection

**Figure 3:**
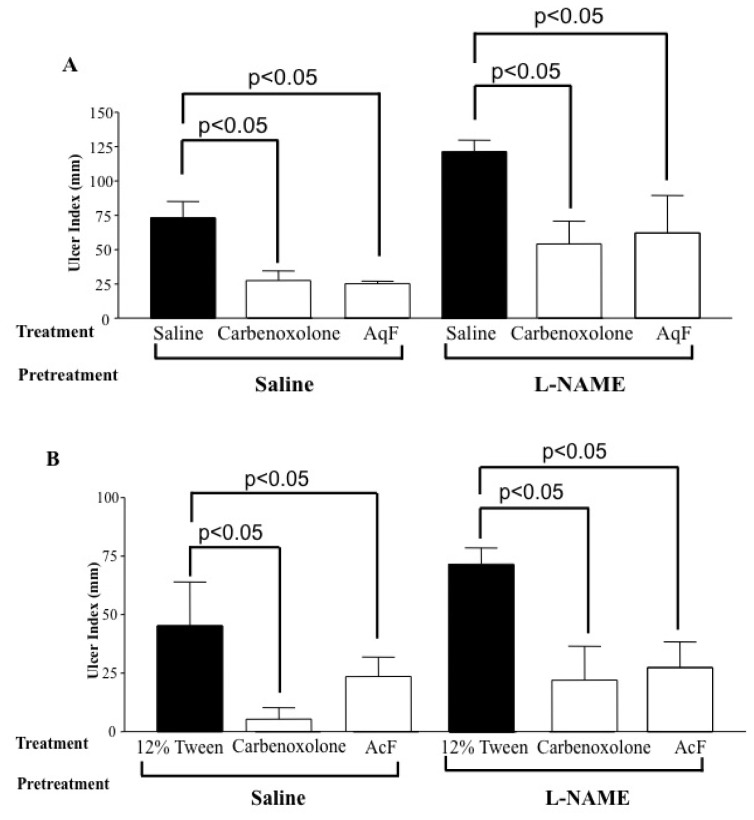
The ulcer index for gastric ulcers induced by ethanol in rats pretreated with L-NAME (70 mg/kg) alone or together with carbenoxolone (100 mg/kg), (**A**) AqF (100 mg/kg) or (**B**) AcF (50 mg/kg) of *Indigofera truxillensis*. The results are reported as the mean ± SD. ANOVA followed by Tukey’s test. *p* < 0.05 compared to the corresponding vehicle group.

**Figure 4:**
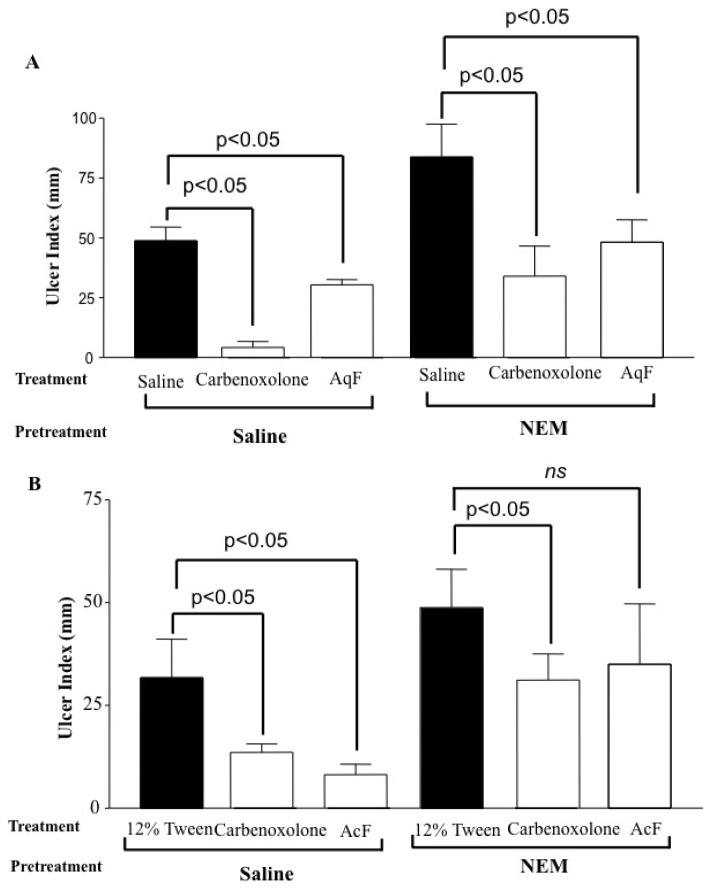
The ulcer index for gastric ulcers induced by ethanol in rats pretreated with *N*-ethylmaleimide (NEM) (10 mg/kg) alone or together with carbenoxolone (100 mg/kg) (**A**) AqF (100 mg/kg) or (**B**) AcF (50 mg/kg) of *Indigofera truxillensis*. The results are reported as the mean ± S.D. ANOVA followed by Tukey’s test. *p* < 0.05 compared to the corresponding vehicle group.

Figure 3 shows that both fractions, AqF (100 mg/kg) and AcF (50 mg/kg), protected the gastric mucosa against ethanol-induced gastric ulcers, even in the L-NAME-challenged animals. When *N*-ethylmaleimide (NEM), an SH blocker, was administered, only the AcF (50 mg/kg) gastroprotection was attenuated (Figure 4A,B).

Recent research has also highlighted the fact that the protective functions of prostaglandins in the stomach can be exerted by other mediators, in particular NO [22]. The release of NO causes vasodilatation of submucosal arterioles increasing the mucosal blood flow. This increase in blood flow allows the buffering of acid that has entered the *lamina propria* and helps to dilute and remove any toxins that have crossed the epithelium [23].

Aiming at evaluating the participation of endogenous nitric oxide (NO) in the gastroprotection of AqF and AcF, we used L-NAME, an NO synthase inhibitor. Our results did not show any increase in the gastric lesions after blocking nitric oxide production with L-NAME, suggesting that the gastroprotective effect of both fractions was not mediated by the nitric oxide pathway.

Non-protein sulfhydryl (SH) compounds limit the production of oxygen-derived free radicals and are involved in cellular protection [24]. In experimental animals, ethanol-induced damage to the gastric mucosa was associated with a significant decrease in the levels of SH-compounds, especially glutathione [25]. SH-compounds have been implicated in the maintenance of gastric integrity, particularly when reactive oxygen species are involved in the pathophysiology of tissue damage [26]. Figure 4 shows that NEM, an SH blocker, significantly attenuates the gastroprotective effect of AcF (50 mg/kg), which indicates that part of the protective action is mediated by endogenous SHs.

### 2.7. Gastric Ischemia–Reperfusion

The ischemia achieved in the different experimental groups by occlusion of the celiac artery for 30 min, followed by 60 min of reperfusion, produced ulcerative lesions in the gastric mucosa of the rats. Table 3 shows a significant reduction of lesions in the animals pretreated with rutin (200 mg/kg) and both fractions AqF (100 mg/kg) and AcF (50 mg/kg) from *I. truxillensis* compared with the respective controls.

Ischemia and reperfusion (IR) are known to induce gastric lesions, predominantly due to the excessive formation of reactive oxygen metabolites that induce inflammatory responses and tissue damage by fragmenting cellular DNA [15]. Ischemia weakens the gastric mucosal barrier and increases the acid back-diffusion predisposing the gastric mucosa to damage [27]. After reperfusion, reactive oxygen species (ROS) are generated from the xanthine-xanthine oxidase system and activate neutrophils, leading to tissue lipid peroxidation, which in combination with gastric secretion results in damage and cellular death [28].

ROS are involved in the ethanol-induced mucosal damage leading to oxidative stress. In order to protect tissues against the damage provoked by ROS, the cells contain antioxidant enzymes, including superoxide dismutase (SOD), glutathione peroxidase (GSH-Px) and glutathione reductase (GSH-Rd) [7].

The gastric lesions were reduced by both AqF and AcF fractions by 82% and 95%, respectively, when compared to the respective control groups. This protection may be due to flavonoid presence in the species challenged. These compounds present antioxidant activity, inhibiting the formation of free radicals [29]. Considering these results, we evaluated which enzymes (SOD, GSH-Px and GSH-Rd) were involved in the antioxidant activity.

### 2.8. Glutathione Peroxidase (GSH-Px), Glutathione Reductase (GSH-Gr) and Superoxide Dismutase (SOD) Activities

In the antioxidant assays we evaluated the enzyme activities of GSH-Px, GSH-Rd and SOD in the ischemia/reperfusion-induced ulcer model. Table 4 shows that pretreatment with AqF (100 mg/kg) significantly increases the SOD and GSH-Rd activities, but does not alter the GSH-Px activity. AcF (50 mg/kg) promotes an increase in GSH-Px, GSH-Rd and SOD activities.

The antioxidant enzyme SOD plays an important role in the protection against oxidative stress [30]. This enzyme is the first gastric mucosa antioxidant enzyme, which catalyzes the dismutation of O_2_^•^ into H_2_O_2_, which is less harmful [8]. The GSH-Px is fundamental to the elimination of hydrogen peroxide and lipid hydroperoxides in the gastric mucosa cells [31]. The antioxidant activity of GSH-Px is coupled with the oxidation of reduced glutathione (GSH), which can subsequently be reduced by GSH-Rd using NADPH as the reducing agent [8]. We observed that pre-treatment with AqF (100 mg/kg) significantly increased the activity of SOD and GSH-Rd. AcF (50 mg/kg) promoted a significant increase in the activity of SOD, GSH-Px and GSH-Rd. ROS participate in the etiology and pathophysiology of gastric ulcers [32]. Drugs with multiple mechanisms of protective action, such as antioxidant properties, may be one way forward towards minimizing tissue injury in human disease [33]. Medicinal plants present many compounds that may modulate the levels of various endogenous antioxidant enzymes [34] and thus can be a useful strategy in the gastric ulcer treatment.

### 2.9. Healing in Acetic-Induced Gastric Lesions

In the acetic acid-induced gastric ulcer model, the 14-consecutive day treatment with cimetidine (100 mg/kg) and AcF from *I. truxillensis* reduced the ulcer area by 68% and 38%, respectively. On the other hand, the AqF (100 mg/kg) fraction was not able to reduce the ulcers induced by acetic-acid (Table 5).

The acetic acid ulcer model highly resembles human ulcers in terms of both pathological features and healing process [35,36].

The ulcer produced by the injection of acetic acid into the stomach wall of the rats is assumed to be similar to the human chronic ulcer, since it is difficult to be treated and it takes long to heal. Studies have demonstrated that reepithelialized mucosa of grossly “healed” experimental gastric ulcers has prominent histologic and ultrastructural abnormalities: reduced height, marked dilation of gastric glands, increased connective tissue, a disorganized microvascular network and increased capillary permeability [37]. These abnormalities may interfere with mucosal defense and cause ulcer recurrence when ulcerogenic factors are present [38].

Postoperative treatment with AqF (100 mg/kg) and AcF (50 mg/kg) for 14 consecutive days demonstrated that only AcF accelerated ulcer healing. On day 14 after surgery, the percentage of rats with cicatrized ulcers in AcF was significantly higher than those of the vehicle group. Ulcer healing is a genetically programmed repair process, which is controlled by growth factors such as EGF [38]. Alkaloid, a major compound in AcF, increased the EFG expression in gastric mucosa in acetic acid-induced gastric lesions [39]. This fact can partially explain the healing enhancement showed by AcF. On the other hand, AqF had no significant effect on the ulcerated area. However, we observed a thicker regenerative mucosa under treatment with AqF, which denotes greater cell proliferation at that site, contributing to the cicatrization of the ulcer [40].

The traditional use of *Indigofera truxillensis* is reported by the pharmacopeia of Cuba, in which the aerial parts of this species are used for digestive tract disorders [2]. Cola-Miranda *et al.*[3] confirmed the activity attributed by folk medicine studying the anti-ulcerogenic activity of *I. truxillensis* that pointed to an antissecretory and cytoprotective mechanism. However, the gastroprotective action does not ensure a healing activity of the plant in an already installed gastric lesion, and this has still not been evaluated. In this context, *I. truxillensis* was evaluated for its efficacy in the ulcer-healing model, which highly resembles human ulcers [35,36] and is effective only with the AcF fraction. Furthermore, both fractions protected the gastric mucosa against damage caused by ROS and increased the key activity of anti-oxidizing enzymes such as superoxide dismutase in the IR model. Thus, this study showed novel healing and antioxidant activities of the species *I. truxillensis*.

## 3. Experimental Section

### 3.1. Animals

Male Unib: WH rats (180–250 g) obtained from the State University of Campinas (CEMIB/UNICAMP) Brazil, were used. The rats were fed a certified Nuvilab CR-diet, with free access to tap water and were housed on a 12 h light/dark cycle at 60 ± 1% humidity and 21.5 ± 2 °C. The experimental protocols were approved by the institutional Committee for Ethics in Animal Experimentation (CEEA/UNICAMP) and were performed in accordance with the Canadian Council Guide lines for Animal Care.

### 3.2. Drugs

The following drugs were used: cimetidine, lansoprazole, carbenoxolone, indomethacin, and *N*-ethyl-maleimide (NEM). All drugs and chemicals used were obtained from Sigma Chemical Co. (St. Louis, MO, USA) and were prepared immediately before use.

### 3.3. Plant Material

The leaves of *I. truxillensis* were collected along the Domingos Sartori highway in Rubião Junior, Botucatu, São Paulo State, Brazil, in June 2003. The plants were identified by Jorge Tamashiro of the Institute of Biology at UNICAMP and a voucher specimen (UEC: 131.827) was deposited in the Herbarium at UNICAMP.

### 3.4. Preparation of Fractions

The leaves (1500 g) of *I. truxillensis* were air dried (seven days at 40 °C), powdered, and then exhaustively extracted with chloroform (CHCl_3_) and methanol (MeOH) successively, at room temperature (three chloroform-methanol cycles, with 72 h for each solvent). The solvents were evaporated in vacuum to provide a CHCl_3_ extract (43 g, 3%) and a MeOH extract (110 g, 7.3%). A portion (5.0 g) of the MeOH extract was partitioned in ethyl acetate and water (1:1, *v*/*v*) to yield 1.6 g (32%) of the ethyl acetate (AcF) and 2.4 g (48%) of the aqueous fraction (AqF).

### 3.5. Identification of Fraction Constituents AcF and AqF

The AcF and AqF fractions were fractionated in analogous ways by gel permeation chromatography. An aliquot of the each fraction (500 mg) was subjected to column chromatography on Sephadex LH-20, using methanol as eluent, flowing at 0.5 mL/min. The collected fractions were combined into three fractions groups after thin layer chromatography (TLC) analysis. Fraction 1 was analyzed by direct injection ESI-IT-MS/MS (electrospray ionization ion trap tandem mass spectrometry), which demonstrated that this fraction contained glycerolipids. Fraction 2, denominated the flavonoid fraction, was purified by column chromatography and afforded flavonol derivatives of kaempferol. Fraction 3, denominated the alkaloid fractions, was chromatographed by Sephadex LH-20 column and exhibited bis-indole alkaloids. Compounds in Fractions 2 and 3 were identified by MS (mass spectrometry), NMR (nuclear magnetic resonance) techniques and confirmed by comparing the physical and spectroscopic/spectrometric data with those in the literature [6].

### 3.6. Antiulcer Activity

#### 3.6.1. Ethanol-Induced Gastric Lesions

Ethanol-induced ulcers were produced in rats according to the method of Morimoto *et al.*[41]. Rats (*n* = 5) were randomly separated into ten groups and fasted for 24 h before the experiment. One hour after the oral administration of AcF or AqF from *I. truxillensis* (25, 50, and 100 mg/kg), lansoprazole (30 mg/kg), 12% Tween 80 or Saline (10 mL/kg), 1 mL of 99.5% ethanol was given orally to the rats. Animals were killed by cervical dislocation 1 h after ethanol administration. Their stomachs were removed, opened along the greater curvature, and fixed between two glass plates. The inner surface of the stomach was examined with a dissecting microscope (Nikon SMZ800) and the number of gastric lesions was counted. The ulcer index was calculated according to the method of Szelenyi and Thiemer [42].

#### 3.6.2. Gastric Secretion in Lesions Induced by Pylorus Ligature

The method of Shay *et al.*[43] was used with some modifications. Rats (*n* = 5) were fasted for 36 h and, immediately after pylorus ligature, AcF (50 mg/kg) and AqF (100 mg/kg) from *I. truxillensis*, cimetidine (100 mg/kg), 12% Tween 80 or Saline (10 mL/kg) was administered intraduodenally. The rats were killed 4 h later, and their abdomens were opened and the stomachs removed. The gastric juice was collected and weighed (g) and its pH was determined using a pH meter (Quimis Aparelhos Científico Ltda, model Q400A, Brazil).

#### 3.6.3. Prostaglandin Production Determination

Male rats were divided into nine groups (*n* = 6). After a 24 h fast, the animals received a pretreatment of saline, s.c. (Groups 1, 2, 3 and 4), or indomethacin (dissolved in 5% sodium bicarbonate solution) 30 mg/kg, s.c. (Groups 5, 6, 7 and 8). The ninth group consisted of the sham control animals. Thirty minutes after pretreatment, saline (Groups 1 and 5), 12% Tween 80 (Groups 2 and 6), AcF 50 mg/kg (Groups 3 and 7) or AqF 100 mg/kg (Groups 4 and 8) was administered orally. Thirty minutes after treatment, all the animals were sacrificed and their abdomens opened. A sample of the corpus (full thickness) was excised, weighed and suspended in 1 mL of 1 mM sodium phosphate buffer, pH 7.4. The tissue was finely minced with scissors and then incubated at 37 °C for 20 min. PGE_2_ in the buffer was measured by the enzyme immunoassay (R & D systems) and the absorbance was read at 450 nm [44].

#### 3.6.4. Determination of the Gastric Mucus Content

This assay was done as described by Rafatullah *et al.*[45] with some modifications. After a 36 h fast, rats (*n* = 5) received AcF (50 mg/kg) and AqF (100 mg/kg) from *I. truxillensis*, carbenoxolone (200 mg/kg), 12% Tween 80 and Saline (10 mL/kg) orally. Thirty minutes after treatment, the pylorus was ligated. The animals were killed by cervical dislocation 4 h after pylorus ligation and the glandular portion of the stomachs was removed and weighed. Each segment was immediately immersed in 10 mL of 0.1% Alcian blue solution (0.16 M sucrose/0.05 M sodium acetate, pH 5.8) for 2 h, after that the excess dye was removed by two successive rinses with 10 mL of 0.25 M sucrose, first for 15 min and then for 45 min. Each stomach was then transferred to 0.5 M magnesium chloride solution for 2 h. Four milliliters of the dye solution was then vigorously shaken with an equal volume of ether and the resulting emulsion was centrifuged at 2000 × *g* and the absorbance of the aqueous layer was measured at 580 nm. The amount of blue dye extracted per gram of wet glandular tissue was then calculated from a standard curve of dye prepared in sucrose-acetate solution.

#### 3.6.5. Determination of the Role of Nitric Oxide (NO) and Sulfhydryl Compounds (SH) in Gastric Protection

Male rats (*n* = 5) were divided into eight groups and pretreated (i.p.) with saline, L-NAME (*N*-nitro-l-arginine methyl ester 70 mg/kg), an inhibitor of the NO synthesis, or NEM (*N*-ethylmaleimide, 10 mg/kg), a blocker of SH compounds [46]. Thirty minutes after the pretreatment, the animals were administered (p.o.) 12% Tween 80 and Saline (10 mL/kg), carbenoxolone (100 mg/kg), AcF (50 mg/kg) or AqF (100 mg/kg). After 60 min, all the groups received 1 mL absolute ethanol to induce gastric ulcers. One hour after receiving ethanol the rats were killed for the determination of gastric lesions.

#### 3.6.6. Gastric Ischemia–Reperfusion

Ischemia–reperfusion damage was produced in rats by a method proposed by Ueda *et al.*[47]. Rats (*n* = 5) received AcF (50 mg/kg) and AqF (100 mg/kg) from *I. truxillensis*, rutin (200 mg/kg), 12% Tween 80 and Saline (10 mL/kg) orally. Thirty minutes after, the animals were anaesthetized by intramuscular injection of Ketamine (50 mg/Kg)/Xylazine (10 mg/Kg). The left side of the abdomen was shaved, and an incision was made. Briefly, the celiac artery was dissected, free of fat excess and clamped for 30 min (ischemia phase) using a micro-bulldog clamp. Reoxygenation was allowed by removing the clamp for 60 min (reperfusion phase). At the end of this period, the animals were sacrificed by cervical dislocation and the stomachs were excised and opened along the great curvature for the detection of the ulcer area. A mucosal scrapping was performed right after for the quantification of the enzymes (SOD, GSH-Px e GSH-Gr).

### 3.7. Enzymatic Assays

For the enzymatic assays, the stomach of each rat was removed, the mucosa was scrapped off using two glass slides on ice, homogenized in phosphate buffer (0.1 M, pH 7.4) and frozen at −80 °C until biochemical determination. The changes in absorbance were determined using a Fusion Packard spectrophotometer, and the results were expressed as pmol/min/mg protein for GSH-Px and GSH-Gr and U/mg protein for SOD activity. The protein concentration of the samples was determined following the method described by Bradford [48]. GSH-Px activity was quantified by following the decrease in absorbance at 365 nm induced by 0.25 mM H_2_O_2_ in the presence of reduced glutathione (10 mM), NADPH, (4 mM), and 1 U enzymatic activity of GSH-Gr [49]. Glutathione reductase activity was measured according to Worthington and Rosemeyer [50], following the decrease in absorbance at 340 nm induced by oxidized glutathione in the presence of NADPH in phosphate buffer, pH 7.8. Absorbance changes were read between 1 and 10 min. SOD activity was analyzed by the reduction of nitroblue tetrazolium using a xanthine-xanthine oxidase system, *i.e.*, superoxide generation [51].

### 3.8. Healing in Acetic-Induced Gastric Lesion

The experiment was done according to the method described by Takagi *et al.*[35] with some modifications Okabe and Amagase [36]. Three groups (*n* = 5) of male Unib: WH rats were fasted for 24 h before this experiment. Under anesthesia, a laparotomy was done in all animals through a midline epigastric incision. After exposing the stomach, 0.05 mL (*v*/*v*) of a 30% acetic acid solution was injected into the subserosal layer in the glandular part of the anterior wall. The stomach was bathed with saline (20 °C) to avoid adherence to the external surface of the ulcerated region. The abdomen was then closed and all the animals were fed normally. We selected the lowest effective dose of both AcF (50 mg/kg) and AqF (100 mg/kg) fractions of *I. truxillensis*; cimetidine (100 mg/kg), 12% Tween 80 and Saline (10 mL/kg), for the determination of the healing effects by the subacute treatment. All treatments were done orally once a day during 14 consecutive days beginning one day after surgery.

One day after the last drug administration, the rats were killed and the stomachs were removed. The gastric lesions were evaluated by examining the inner gastric surface with a dissecting magnifying glass. The macroscopic ulcer area (mm^2^) and curative ratio (%) were subsequently determined as described by Takagi *et al.*[35].

### 3.9. Statistical Analysis

The results were expressed as the mean ± standard deviation. Statistical comparisons were done by one-way analysis of variance (ANOVA) followed by the Dunnett’s or Tukey’s post hoc test, with the level of significance set at *p* < 0.05.

## 4. Conclusions

In conclusion, *I. truxillensis* showed antiulcerogenic activity. Prostaglandin production, mucus secretion and antioxidant enzymes are involved in the gastroprotection exerted by this species, probably due to the metabolites present in the plant.

## Figures and Tables

**Table 1:** Effects of lansoprazole (30 mg/kg), aqueous fraction (AqF) and ethyl acetate (AcF) from *Indigofera truxillensis* (50, 100 and 200 mg/kg) on ethanol-induced gastric mucosal ulcers in rats. The results are reported as the mean ± SD. ANOVA followed by Dunnett’s test.

Treatments (p.o.)	*N*	Dose (mg/kg)	Ulcer index	Inhibition (%)
Saline	5	10 mL/kg	66.4 ± 15.4	-
Lanzoprazole	5	30	19.8 ± 6.4 **	70
AqF–*I. truxillensis*	5	50	43.4 ± 14.6 *	35
AqF–*I. truxillensis*	5	100	13.8 ± 6.3 **	79
AqF–*I. truxillensis*	5	200	25.4 ± 9.0 **	61

12% Tween	5	10 mL/kg	33.8 ± 13.1	-
Lansoprazole	5	30	1 ± 0.7 **	97
AcF–*I. truxillensis*	5	50	6.4 ± 2.7 **	81
AcF–*I. truxillensis*	5	100	6.2 ± 4.0 **	81
AcF–*I. truxillensis*	5	200	6.0 ± 2.5 **	82

* *p* < 0.05,

** *p* < 0.01 compared to the corresponding vehicle group.

**Table 2:** Effects of intraduodenal adminstration of cimetidine (100 mg/kg) AqF (100 mg/kg) and AcF (50 mg/kg) from *Indigofera truxillensis* on the biochemical parameters of gastric juice in pylorus-ligated rats. The results are the mean ± SD of five rats per group. ANOVA followed by Dunnett’s test.

Treatments (i.d.)	*N*	Dose (mg/kg)	pH units	Gastric juice (mg)
Saline	5	10 mL/kg	2.7 ± 0.2	0.5 ± 0.1
Cimetidine	5	100	3.2 ± 0.1 **	0.5 ± 0.0
AqF–*I. truxillensis*	5	100	2.8 ± 0.1	0.5 ± 0.1

12% Tween	5	10 mL/kg	3.2 ± 0.1	0.6 ± 0.1
Cimetidine	5	100	3.7 ± 0.2 **	0.5 ± 0.1
AcF–*I. truxillensis*	5	50	3.3 ± 0.2	0.4 ± 0.1 *

* *p* < 0.05,

** *p* < 0.01 compared to the corresponding vehicle group.

**Table 3:** Effect of orally administered AqF (100 mg/kg) and AcF (50 mg/kg) from *Indigofera truxillensis* on ulcers produced by ischemia-reperfusion in rats. The results are reported as the mean ± SD. ANOVA followed by Tukey’s test.

Treatment (p.o)	*N*	Dose (mg/kg)	Ulcer area (mm^2^)	Inhibition (%)
Sham	5	-	0 ± 0 **	-
Saline	5	10 mL/kg	1.7 ± 0.8	-
Rutin	5	200	0.3 ± 0.1 **	82
AqF–*I. truxillensis*	5	100	0.3 ± 0.1 **	82
12% Tween	5	10 mL/kg	15.6 ± 5.3	-
Rutin	5	200	5.3 ± 3.2 **	66
AcF–*I. truxillensis*	5	50	0.8 ± 0.1 **	95

** *p* < 0.01 compared to the corresponding vehicle group.

**Table 4:** Effect of AqF (100 mg/kg) and AcF (50 mg/kg) on antioxidant enzymes activity in the gastric mucosa of rats submitted to ischemia-reperfusion. The results are reported as the mean ± SD. ANOVA followed by Dunnett’s test.

Treatment	Dose (mg/kg)	SOD (U/mg of protein)	GSH-Px (pmol/min/mg of protein)	GSH-Rd (pmol/min/mg of protein)
Sham	-	10.6 ± 1.9	36.9 ± 8.7	27.6 ± 2.8
Saline	10 mL/kg	3.7 ± 0.5	23.8 ± 1.5	12.8 ± 0.1
Rutin	200	19.0 ± 4.3 **	19.2 ± 4.0	19.3 ± 3.2
AqF–*I. truxillensis*	100	8.8 ± 0.9 ^*^	26.5 ± 3.9	109.2 ± 28.0 **

12% Tween	10 mL/kg	3.8 ± 0.7	13.9 ± 0.9	8.6 ± 0.5
Rutin	200	7.8 ± 1.5	16.2 ± 1.6	15.2 ± 2.4
AcF–*I. truxillensis*	50	8.9 ± 1.9 ^*^	33.7 ± 5.6 ^*^	22.4 ± 3.5 ^*^

** *p* < 0.01 compared to the corresponding vehicle group.

**Table 5:** Effect of orally administered AqF (100 mg/kg) and AcF (50 mg/kg) from *Indigofera truxillensis* on healing of ulcers produced by the injection of a 30% acetic acid solution into the stomachs of rats. The ulceration was scored on the 15th day after surgery. The results are reported as the mean ± SD. ANOVA followed by Dunnett’s test.

Treatment	*N*	Dose (mg/kg)	Ulcer area (mm^2^)	Cure rate (%)
Sham	5	-	0 ± 0 **	-
Saline	5	10 mL/kg	3.9 ± 0.9	-
Cimetidine	5	100	1.6 ± 0.6 **	57
AqF–*I. truxillensis*	5	100	3.6 ± 0.8	-

12% Tween	5	10 mL/kg	4.5 ± 0.4	
Cimetidine	5	100	1.4 ± 0.5 **	68
AcF–*I. truxillensis*	5	50	2.8 ± 1.4 *	38

* *p* <0.05,

** *p* < 0.01 compared to the corresponding vehicle group.
